# *Drosophila melanogaster* positive transcriptional elongation factors regulate metabolic and sex-biased expression in adults

**DOI:** 10.1186/s12864-017-3755-x

**Published:** 2017-05-18

**Authors:** Haiwang Yang, Denis Basquin, Daniel Pauli, Brian Oliver

**Affiliations:** 1 National Institute of Diabetes and Digestive and Kidney Diseases, National Institutes of Health, 50 South Drive, Bethesda, MD 20892 USA; 2 Department of Genetics & Evolution, Sciences III, University of Geneva, Boulevard d’Yvoy 4, CH 1205 Geneva, Switzerland

**Keywords:** *lilli*, AFF4, *Su(Tpl)*, ELL, *Cdk9*, P-TEFb, SEC, Pausing

## Abstract

**Background:**

Transcriptional elongation is a generic function, but is also regulated to allow rapid transcription responses. Following relatively long initiation and promoter clearance, RNA polymerase II can pause and then rapidly elongate following recruitment of positive elongation factors. Multiple elongation complexes exist, but the role of specific components in adult Drosophila is underexplored.

**Results:**

We conducted RNA-seq experiments to analyze the effect of RNAi knockdown of *Suppressor of Triplolethal* and *lilliputian.* We similarly analyzed the effect of expressing a dominant negative *Cyclin-dependent kinase 9* allele. We observed that almost half of the genes expressed in adults showed reduced expression, supporting a broad role for the three tested genes in steady-state transcript abundance. Expression profiles following *lilliputian* and *Suppressor of Triplolethal* RNAi were nearly identical raising the possibility that they are obligatory co-factors. Genes showing reduced expression due to these RNAi treatments were short and enriched for genes encoding metabolic or enzymatic functions. The dominant-negative *Cyclin-dependent kinase 9* profiles showed both overlapping and specific differential expression, suggesting involvement in multiple complexes. We also observed hundreds of genes with sex-biased differential expression following treatment.

**Conclusion:**

Transcriptional profiles suggest that Lilliputian and Suppressor of Triplolethal are obligatory cofactors in the adult and that they can also function with Cyclin-dependent kinase 9 at a subset of loci. Our results suggest that transcriptional elongation control is especially important for rapidly expressed genes to support digestion and metabolism, many of which have sex-biased function.

**Electronic supplementary material:**

The online version of this article (doi:10.1186/s12864-017-3755-x) contains supplementary material, which is available to authorized users.

## Background

Transcription by eukaryotic RNA polymerase II complex (RNAPII), composed of ten or more subunits [1, 2], involves multiple steps including initiation, promoter clearance, elongation, and termination. Each step requires different activities. The initiation process involves RNAPII recognition of core promoter elements at the transcription start site (TSS) [3]. Bound RNAPII opens a bubble in the duplex DNA and begins transcribing short RNAs by a scrunching mechanism [4]. While the first steps of initiation and promoter clearance are slow and highly regulated, elongation is rapid [5, 6]. This makes elongation an ideal mechanism for rapidly responding to changing conditions. A stalled or paused RNAPII has completed the slow steps such that de-pausing allows for rapid completion of transcription.

Great progress has been made on the biochemistry of elongation, although the exact composition of complexes is unclear due to biological context, biochemical complexity, and/or methodologies used in various studies. For the majority of genes, after transcription of about 20 to 50 nucleotides, RNAPII pauses when 5,6-dichloro-1-β-D-ribofuranosylbenzimidazole (DRB) sensitivity inducing factor (DSIF, a heterodimer of Suppressor of Ty 4 and 5 (Spt4 and Spt5)) and negative elongation factor (NELF) protein complexes bind to the RNAPII and transcript [7–9], to form a DSIF-NELF complex promoting the pausing of RNAPII [10–12]. Paused RNAPII is released by the positive transcription elongation factor b complex (P-TEFb), which phosphorylates the DSIF and NELF complexes as well as the carboxy-terminal domain of RNAPII (CTD) [13–17]. The catalytic and regulatory components of P-TEFb are *Cyclin-dependent kinase 9* (*Cdk9*) and *Cyclin T* respectively [18–20]. In addition to P-TEFb, *Cdk9* can also form a complex with *Cyclin K* [21]. Recruitment of P-TEFb kinase dissociates NELF, transforming DSIF into a positive elongation factor, triggering elongation [10].

P-TEFb is often part of larger complexes (Table 1). For example, the super elongation complex (SEC), is a key regulator required for transcriptional elongation checkpoint control and rapid transcriptional induction [22–24]. In addition to P-TEFb, SEC contains 11-19 Lysine-rich Leukemia (ELL), encoded by *Suppressor of Triplolethal* (*Su(Tpl)*), and ALL1-Fused Gene From Chromosome 4(AF4)/Fragile mental retardation 2 (FMR2) family member 4 (AFF4), encoded by *lilliputian* (*lilli*) [23, 25, 26]. Another transcriptional elongation complex is the Dot1 complex (DotCom) [27], which contains the histone H3 Lys 79 (H3K79) methyltransferase DOT1L, encoded by* grappa* (*gpp*) [28]. H3K79 methylation by DOT1 may regulate transcription of genes by changing chromatin structure and accessibility of transcription factors [29], and has been implicated in many basic functions such as cell cycle regulation, chromosomal stability, and the DNA damage response [30]. There is also evidence that *lilli* and *Su(Tpl)* are essential for the H3K79 methylation function of DotCom during transcriptional activation, and evidence of large complexes including components of DOT1, P-TEFb, and SEC, such as the Elongation Assisting Protein complexes (EAP) and complexes of Mixed Lineage Leukemia 1 proteins (MLL) [23, 27, 31, 32]. Additional complexity includes the Little Elongation Complex (LEC), which contains Su(Tpl) but lacks Lilli and Cdk9 [33], and AF4 family/ENL family/P-TEFb complex (AEP), which contains Lilli and Cdk9 but lacks Su(Tpl) [34]. These studies indicate that there are many possible elongation complexes [25, 31]. We have explored the roles of these complexes by examining the RNA expression profiles when some of these key components are altered in Drosophila. Specifically, knockdown of *lilli* or *Su(Tpl)* should disrupt SEC, EAP, and MLL. LEC should be altered by knockdown of *Su(Tpl)* but not *lilli*, and AEP should be altered by knockdown of *lilli* but not *Su(Tpl)*. The dominant-negative form of *Cdk9* should disrupt P-TEFb, SEC, and AEP, but not MLL.

**Table 1:** Components of different elongation related complexes or protein group

Complex^a^	Lilli or AFF4	Su(Tpl) or ELL	Cdk9	Other components	References
P-TEFb	-^b^	-	+	Cyclin-T, Cyclin-K	[17, 93, 94]
SEC	+	+	+^c^	AFF1, AF9, EAF, ENL	[23, 27]
DotCom	-	-	-	AF9, ENL, Dot1	[27, 95]
EAP	+	+	+	AF5q31, AF9, ENL, Dot1	[27, 32, 96]
MLL	+	+	-	AFF1, AF5q31, AF9, ENL	[23, 97–99]
LEC	-	+	-	EAF	[24, 33]
AEP	+	-	+	AF5q31, LAF4, ENL	[34]

^a^P-TEFb = Positive Transcript Elongation Factor b; SEC = Super Elongation Complex; DotCom = Dot1 Complex; EAP = Elongation Assisting Proteins; MLL = Mixed Lineage Leukemia 1 proteins; LEC = Little Elongation Complex; AEP = AF4 family/ENL family/P-TEFb complex

^b^“+” and “-” denote inclusion and exclusion of proteins respectively

^c^A few studies regard P-TEFb as an independent component of SEC [27, 100]

The regulatory advantage of RNAPII pausing and elongation control is that rapid responses can be achieved if earlier rate-limiting steps occur, leaving only the more rapid elongation step for completion of transcription [5, 35–37]. Such a rapid transcriptional response is critical when organisms experience rapid environmental changes. For example, RNAPII pausing has been extensively studied in the context of the rapid transcription of Drosophila heat shock genes [38–40]. The rapid response is essential because failure to adjust to rapid temperature change results in catastrophic denaturation or mis-folding of proteins [41]. Increased levels of protein chaperones encoded by the heat shock loci ameliorate this stress. The presence of DSIF and NELF results in RNAPII pausing near the heat shock gene promoters [39], but following a heat shock, P-TEFb quickly redistributes to the heat shock genes, which allows RNAPII to elongate resulting in rapid production of transcripts [42, 43]. Other types of stress requiring a quick response in Drosophila, such as arbovirus infection, are also regulated at the level of elongation [44]. Regulation of transcription elongation also plays a role during development. An example is the pausing of RNAPII downstream of the * sloppy-paired-1* promoter by NELF during the rapid blastoderm-stage of development [45]. It is likely that there are other genes responding rapidly due to normal physiology. Our expression profiles might help identify them.

Elongation control also plays a role in sexual development in Drosophila. Sex is determined by counting X chromosomes [46]. In wild-type flies, XX individuals are female and XY individuals are male. The genic imbalance created by X chromosome monosomy in males is compensated by increasing X chromosome gene expression [47]. At the regulatory level, the XX karyotype results in the activation of *Sex-lethal* (*Sxl*), which controls a major branch in the sex determination network [48]. *Sxl* regulates the splicing of *transformer* (*tra*) pre-mRNA, which ultimately is responsible for nearly all sexually dimorphic aspects of development, physiology, and behavior [49–51]. One of the most important genes downstream of *tra* is *doublesex* (*dsx*), which encodes sex-specific isoforms of a doublesex-mab3-related-transcription factor (DMRT). DMRTs are used to regulate sexual dimorphism in most metazoans [52]. The *lilli* and *Su(Tpl)* loci are likely to be direct targets of Dsx, and while the *Cdk9* locus was not among the most obvious Dsx target genes, it has a strong and conserved Dsx binding site [51, 53]. Knocking down *lilli* or *Su(Tpl)* in *dsx* expressing cells results in dramatic sex- and context-specific phenotypes. For example, in males, the formation of specific first leg bristles known as sex combs, shows female pigmentation and narrower female-like morphology, but male distal/proximal orientation. In females, the first legs are normal, but bristles on the genitalia are missing. In males, the genitalia are normal. Thus, these genes function in female-specific development in one tissue and male-specific development in another [51]. If regulators of elongation play a post-developmental role in other aspects of sexual dimorphism, such as physiology, then we would expect to see sex-specific effects of disrupting the function of those complexes in adult flies.

## Results

### Knockdown and overexpression efficiency

**Fig. 1:**
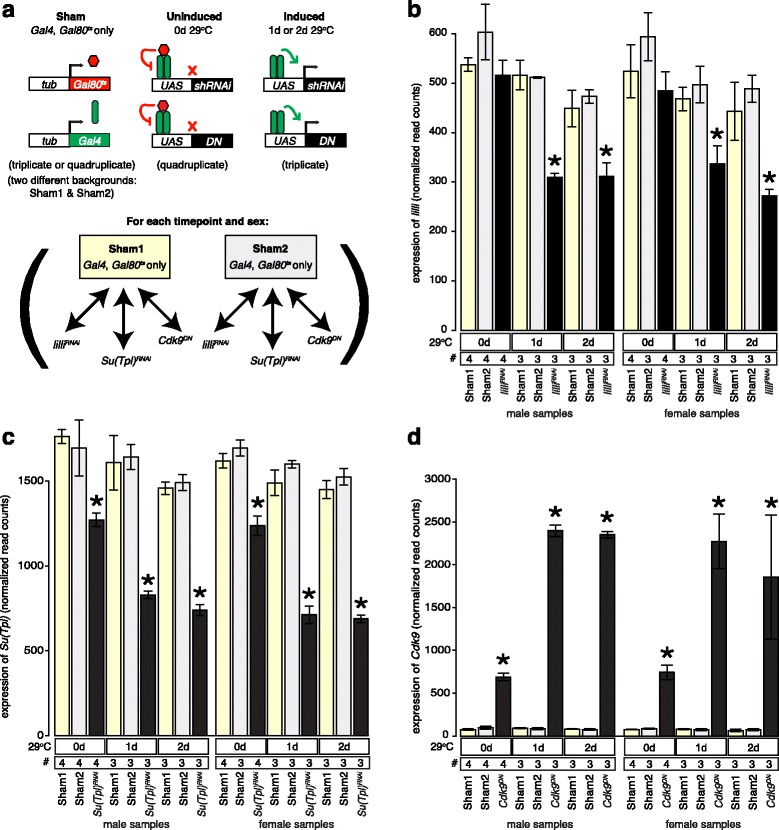
Experimental design and efficiency of RNAi or *Cdk9* ^*DN*^ expression. **a** Experimental design. The cartoons (*top*) illustrate the sham genotypes where Gal80^ts^ (*red*) and Gal4 (*green*) are produced in the absence of a responding UAS transgene (*left*), or when the UAS transgenes are present at the uninducing (*middle*) or inducing (*right*) temperatures. We used homozygous *P{tubP-GAL4}* ^*LL7*^, *P{tubP-GAL80* ^*ts*^}^*7*^ virgin females for all crosses. Sham flies were produced by crossing to *lacZ* (Sham1, *yellow fill*) or *w* ^*1118*^ (Sham2, *gray fill*) males. The schemata (*bottom*) shows how the shams were used as references for all induction timepoints for both females and males in results presented in subsequent figures. See Methods for further information. Transgenes are shown as bars, with regulatory sequences (open) from *tubulin* (*tub*) or the *Gal4 upstream activation sequence* (*UAS*), and encoding sequences (filled) from the *Gal80* ^*ts*^ (*red*), *Gal4* (*green*), *shRNAi*, and *Cdk9* ^*DN*^ (*black*) labeled. Gal80^t*s*^ (*red hexagons*), Gal4 (*green ovals*) proteins are shown*.* Active (*bent black arrows*) and repressed (*red X*) transcription are indicated. **b**-**d** Histograms showing expression of *lilli* (**b**), *Su(Tpl)* (**c**), and *Cdk9* (**d**) transcripts based on normalized read counts (linear scale from DESeq2 [62]) across the gene models after zero (*0d*), one (*1d*)﻿,﻿ or two (*2d*) days of induction. Biological replicate numbers (*#*) are indicated. Values for females (*left*) and males (*right*) are shown in each panel. Knockdown expression levels following induction of *lilli* ^*RNAi*^, *Su(Tpl)* ^*RNAi*^, and *Cdk9* ^*DN*^ (*black*), and shams (*yellow or gray*) as well as one standard deviation (*bars*) are shown. Significant differential expression (*p* adj < 0.05 from DESeq2) relative to sex and timepoint matched shams are shown (*asterisk*)

We were interested in measuring the effect of *lilli*, *Su(Tpl)*, and *Cdk9* on gene expression in adults to probe for roles of elongation. Because these genes are required for organismal viability during development [54, 55], we bypassed the developmental defects by using ubiquitously expressed *Gal4* [56] along with ubiquitously expressed temperature sensitive *Gal80* repressor (*Gal80* ^*ts*^) [57] and *Gal4* responsive constructs expressing RNAi against *lilli*, and *Su(Tpl)* [58] (Fig. 1a). We also used a dominant negative *Cdk9* construct (*Cdk9* ^*DN*^) made by substituting an asparagine for an aspartic acid codon at position 199 (D199N) in the active site of the kinase. The same substitution has been used in other studies, where it has been shown to block P-TEFb CTD kinase activity [59, 60]. *Cdk9* ^*DN*^ under UAS control was introduced into flies using P-element mediated transformation. Expressing *Cdk9* ^*DN*^ during development resulted in lethality (DB and DP, unpublished data). Flies bearing the *Cdk9* ^*DN*^ transgene were viable to adulthood at 20 °C in the presence of both *Gal4* and *Gal80* ^*ts*^.

We were concerned that interfering with these important transcriptional regulators would result in cell lethality and an uninformative signature of dying cells even if we bypassed developmental defects. To assess the effect of these genes on adult viability, we shifted 3 to 4 day old adult flies from the permissive temperature of 20 °C to the restrictive temperature of 29 °C to induce transgene expression. We observed no overt lethality after up to a week. The lack of adult lethality allowed us to measure the effect of *Cdk9, lilli*, and *Su(Tpl)* on the adult steady-state transcriptome in the absence of developmental defects or reduced viability. We crossed *Gal4* and *Gal80* ^*ts*^ to the experimental lines and to two control lines, which acted as shams to monitor any differential expression due to *Gal4* and *Gal80* ^*ts*^ alone (Fig. 1a). We used these sham genotypes without UAS transgenes as controls in addition to the temperature shifts. We collected sexed samples zero, one, and two days post temperature shift to 29 °C. Flies were the same age post-eclosion (7 days) when harvested. Because germline gene expression is not controlled in the expression system we used [61], we removed the gonads from the flies before preparing polyA+ RNA for stranded RNA-seq expression profiling. All experiments were biologically triplicated to quadruplicated using three flies per sample. We used a total of 99 samples, after confirming that mismatched replicate numbers did not impact conclusions (by bootstrapping, see Methods). We obtained above background RNA-seq reads for more than 8000 genes in each sample. We used the DESeq2 [62] Normalized Read Count statistical model to measure differential expression (see Methods). For each experimental sample, we made ratiometric measurements comparing replicate experimental samples to replicate sham samples as a reference. See Methods and GEO [63] (GSE77492) for detailed protocols, data, and replication statistics. A summary of all gene expression data can be found in the Additional file 1.

We first assayed the effectiveness of the adult induction experiments (Fig. 1b to d). We detected expression of *Gal4* and *Gal80* ^*ts*^ in all samples. Prior to the temperature shift, we observed no significant differential expression in *lilli* relative to shams (Fig. 1b), but we did observe modest but significant (Wald test, *p* adj < 0.05 is used throughout) reductions in target gene expression in the *Su(Tpl)* ^*RNAi*^ flies (Fig. 1c). We also observed modest but significant overexpression of *Cdk9* ^*DN*^ (Fig. 1d), and we confirmed that the increased expression was from the transgenic *Cdk9* ^*DN*^ allele by measuring the fraction of the reads bearing the codon substitution (~84% at 20 °C). These data indicate that the *Su(Tpl)* ^*RNAi*^ and *Cdk9* ^*DN*^ transgenes were not fully “off” at 20 °C. Following the temperature shift to 29 °C, we observed dramatic reduction of *Su(Tpl)* (Fig. 1c) and *lilli* (Fig. 1b) steady-state expression in the appropriate RNAi bearing flies and dramatic overexpression of *Cdk9* ^*DN*^ (~96% from the DN allele; Fig. 1d) relative to shams. These data indicate that we altered the expression levels (and the encoded activity in the case of *Cdk9*) of the three genes under study.

### Effects on gene expression

**Fig. 2:**
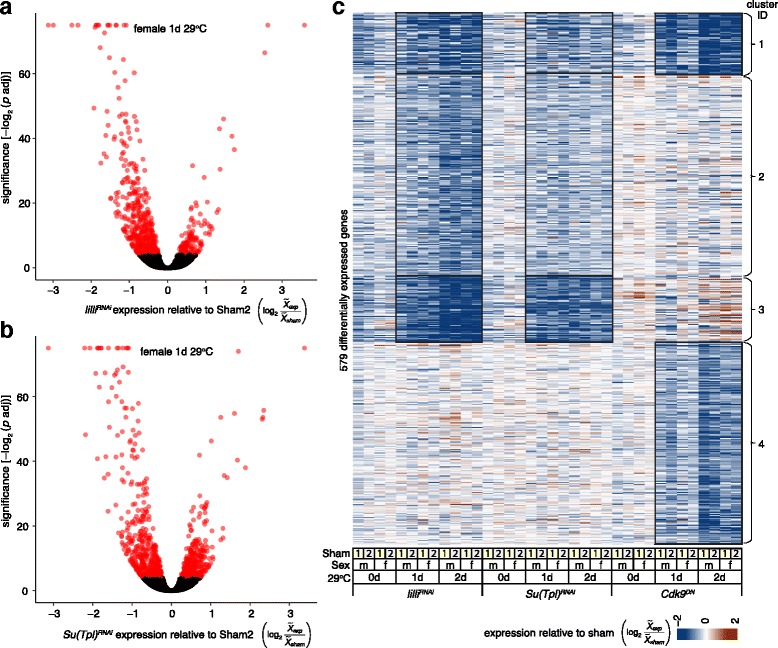
Differential expression and clustering. **a**-**b** Vocano-plots showing examples of differential expression for *lilli* ^*RNAi*^ (**a**) and *Su(Tpl)* ^*RNAi*^ (**b**) one day after induction in female samples relative to Sham2 versus significance (truncated at > 75). Genes with significant differential expression (DESeq2 *p* adj < 0.05, *red*) and no significant differential expression (DESeq2 *p* adj ≥ 0.05, *black*) are shown. Gene expression are shown as log_2_ratios of medians (*X tilde*) of experimental (*exp*) replicates divided by sham replicates. **c** Differential expression heatmap of experimental samples relative to shams. Values indicated in the key (*bottom*) were truncated at +/- 2. Reduced (*blue*) and increased (*red*) expression in experimental samples relative to sham are shown. 579 genes (*rows*) with coherent differential expression relative to shams are shown. Sample comparisons (*columns*) relative to Sham1 (*yellow fill*) or Sham2 (*gray fill*) are indicated (*bottom*), as are female ﻿(*f*) ﻿and male﻿ (*m*)﻿ sex, timepoint in day(s) (*d*), and tested transgenes. The k-means cluster ID used in the text is shown (*right*) and highlighted within the heatmap (*open black boxes*)

To explore the transcriptional responses to transgene induction, we compared replicated results from experimental samples relative to each of the two types of sham samples and asked for significantly differentially expressed genes in any of these time- and temperature-matched comparisons. We observed extensive and significant differences in the gene expression profiles relative to sham controls in these 36 pairwise comparisons, especially for reduced expression in the experimental samples (e.g., Fig. 2a, b). Overall we observed that 4792 genes showed significant differential expression in at least one pairwise comparison. This constituted 27% of all genes in the genome and 58% of the genes that we scored as expressed in all samples. To identify patterns that we could use to classify the responses of this large set of differentially expressed genes in a way that would allow us to infer which elongation complexes might be affected, we used k-means to cluster the expression ratios of experimental relative to sham samples. We removed clusters driven by background genotypes (see Methods). We observed that 579 differentially expressed genes were clearly attributed to *lilli* ^*RNAi*^, *Su(Tpl)* ^*RNAi*^, or *Cdk9* ^*DN*^ (Fig. 2c) treatments. There were four clusters of genes that were dominated by reduced gene expression on treatment: cluster 1 was down-regulated by *lilli* ^*RNAi*^, *Su(Tpl)* ^*RNAi*^ and *Cdk9* ^*DN*^, clusters 2 and 3 were down-regulated following *lilli* ^*RNAi*^ or *Su(Tpl)* ^*RNAi*^ (cluster 3 genes were often up-regulated in *Cdk9* ^*DN*^ flies), and cluster 4 was down-regulated only following *Cdk9* ^*DN*^ expression. There was strong concordance between the *lilli* ^*RNAi*^ and *Su(Tpl)* ^*RNAi*^ profiles in all clusters. That there were no clear clusters of genes differentially expressed due to only one of these RNAi treatments, suggests that wild-type Lilli and Su(Tpl) positively regulate (directly or indirectly) many of the same genes. This is consistent with the hypothesis that these proteins are often obligatory components of the same functional complex. Cluster 1 showed reduced expression following *Cdk9* ^*DN*^ induction, suggesting that wild-type *lilli*, *Su(Tpl)*, and *Cdk9* positively regulate these genes (directly or indirectly), consistent with the participation of all three activities at a subset of genes. This is the correlation expected for disrupted SEC or EAP. Most gene expression altered by *lilli* and *Su(Tpl)* (cluster 2 and 3) were not regulated by *Cdk9*, which is consistent with the composition of MLL. The *Cdk9* ^*DN*^ expression profile was distinct (cluster 4). Therefore, there is also *Cdk9*-specific differential expression profile, consistent with a role for P-TEFb alone.

### Expression of genes encoding enzymes

**Fig. 3:**
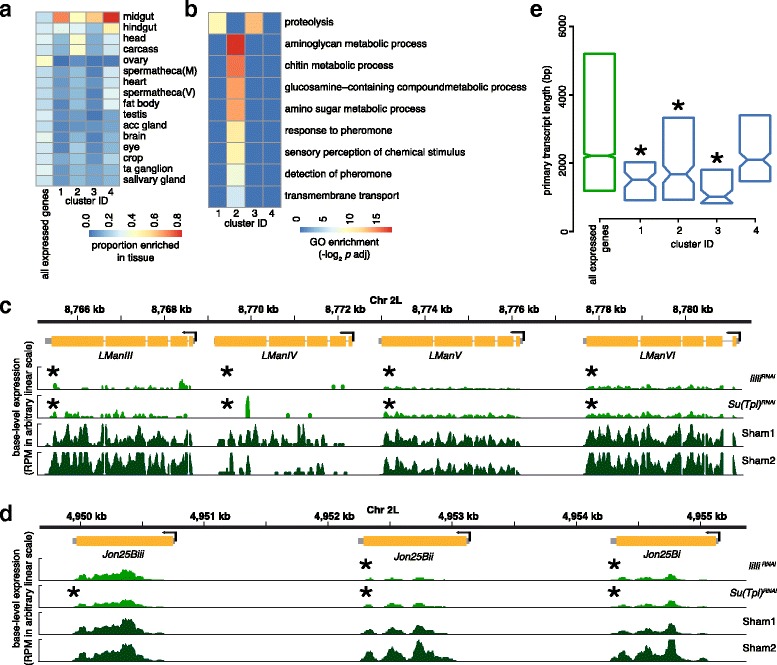
Characteristics of differentially expressed genes. **a** Heatmap showing enrichment for genes expressed in tissues (*rows*) of wild-type flies from FlyAtlas among genes in the Fig. 2 clusters or among all genes expressed in our study (*columns*). The proportion of genes in a column enriched in each tissue (see key) is shown in hierarchical order. Tissues are written out except: ta ganglion = thoracicoabdominal ganglion; CNS = central nervous system; acc gland = male accessory gland; V = virgin, and M = mated. The carcass is the thorax and abdomen without gut and reproductive tract. **b** Heatmap showing GO biological process terms (*rows*) enriched in any cluster (see key). GO terms are also presented in hierarchical clustering order. **c**-**d** Browser views showing expression of two sets of related genes that are genomic neighbors and are positively regulated by *lilli* and *Su(Tpl)* (female, one day after induction). **c** The *lysosomal α-mannosidase* (*LMan*) genes. **d** The *Jonah* (*Jon*) chymotrypsin genes. Chromosome arm and genome coordinates are shown (*top*). The longest annotated transcript of each gene is shown. In the gene models, untranslated (*gray boxes*) and coding (*yellow boxes*) regions, and introns (*gray lines*) are shown. Transcription direction is shown (*bent arrows*). Base-level read density per million reads (RPM) (see Methods; arbitrary scale) is shown for RNAi (*light green*) and sham (*dark green*) as are test genes or sham abbreviations (*right*). Instances of significant induction-dependent differential expression are indicated (*asterisks*). **e** Boxplot of primary transcript length (in base pairs) by cluster (*blue*) or among all expressed genes (*green*). Clusters with significantly shorter (Wilcoxon rank test, *p* < 0.05) primary transcript than all expressed genes are indicated (*asterisks*)

To help determine if positive regulators of transcriptional elongation affect specific tissues or organs, we used FlyAtlas data from adults [64] to determine if differentially expressed genes had enriched expression in particular tissues. We found that the overall expression profile was not strongly enriched for genes expressed in any adult tissues (leftmost column of Fig. 3a), consistent with our use of the entire fly other than the gonads. Strikingly, the genes showing differential expression following *lilli* ^*RNAi*^, *Su(Tpl)* ^*RNAi*^, or *Cdk9* ^*DN*^ induction showed coherence in implied tissue-specificity (Fig. 3a). We found that all clusters with differentially expressed genes had enriched expression in the midgut. This is intriguing, as the midgut environment is subject to rapid changes as food passes through the digestive tract.

To determine if there was a functional category of genes differentially expressed due to the transgenes, we performed Gene Ontology (GO) term analysis (Fig. 3b) using expressed genes as the reference, and selected all terms that were enriched in one of the clusters. The enriched terms were associated with proteolysis, various aspects of amino acid or carbohydrate metabolism, transport, and pheromones. The genes regulated by all three of the transgenes (clusters 1) were enriched for genes encoding typsins with serine-type endopeptidase function (e.g., *Serine protease 6*). The genes down-regulated following *lilli* ^*RNAi*^ or *Su(Tpl)* ^*RNAi*^ but not *Cdk9* ^*DN*^ induction (clusters 2 and 3) were also enriched in genes encoding proteolysis functions in addition to metabolic, sensory, and transport functions. We show two examples of gene groups encoding important digestive enzymes that were down-regulated by transgene induction: the *Lysosomal alpha-mannosidase* (*LMan*) genes (Fig. 3c) and chymotrypsin encoding *Jonah* genes (Fig. 3d). We found that these genes required *lilli* and *Su(Tpl)* (but not *Cdk9*) for high level expression (clusters 2 and 3). They also both showed strongly midgut-biased expression in wild-type flies [64], and their wild-type functions involve carbohydrate hydrolysis in mannose production [65] and proteolysis (serine-type endopeptidase activity) [66] respectively.

We noticed that many of these enzyme-encoding genes with differential expression had short primary transcripts. Therefore, we determined if there was an overall tendency for preferential regulation of genes with short transcripts, by plotting the distributions of annotated primary transcript length of genes in each expression ratio cluster (Fig. 3e). Interestingly, the genes regulated by all three tested genes (cluster 1, see Fig. 2c) have significantly shorter primary transcripts than all expressed genes (1517 versus 2217 bp; Wilcoxon rank test, *p* = 4 × 10^−6^). Moreover, the genes regulated by *lilli* and *Su(Tpl)* (cluster 2 and 3, see Fig. 2c) also have significantly shorter primary transcripts than all expressed genes (1679 and 1027 versus 2217 bp; Wilcoxon rank test, *p* ≤ 8 × 10^−6^). In contrast, the genes regulated by *Cdk9* had insignificantly shorter primary transcript length compared to all expressed genes (2099 versus 2217 bp; Wilcoxon rank test, *p* = 0.6). It is worth noting that the two clusters with the shortest primary transcripts (cluster 1 and 3) also had the strongest down regulation of expression after induction (Fig. 2c), as well as concordant enrichment of genes with proteolysis function (Fig. 3b). Given that control by elongation and short transcript length both contribute to rapid production of mature transcripts, these data suggest that many genes involved in metabolism have been optimized for rapid elongation.

### Sex-biased regulation

**Fig. 4:**
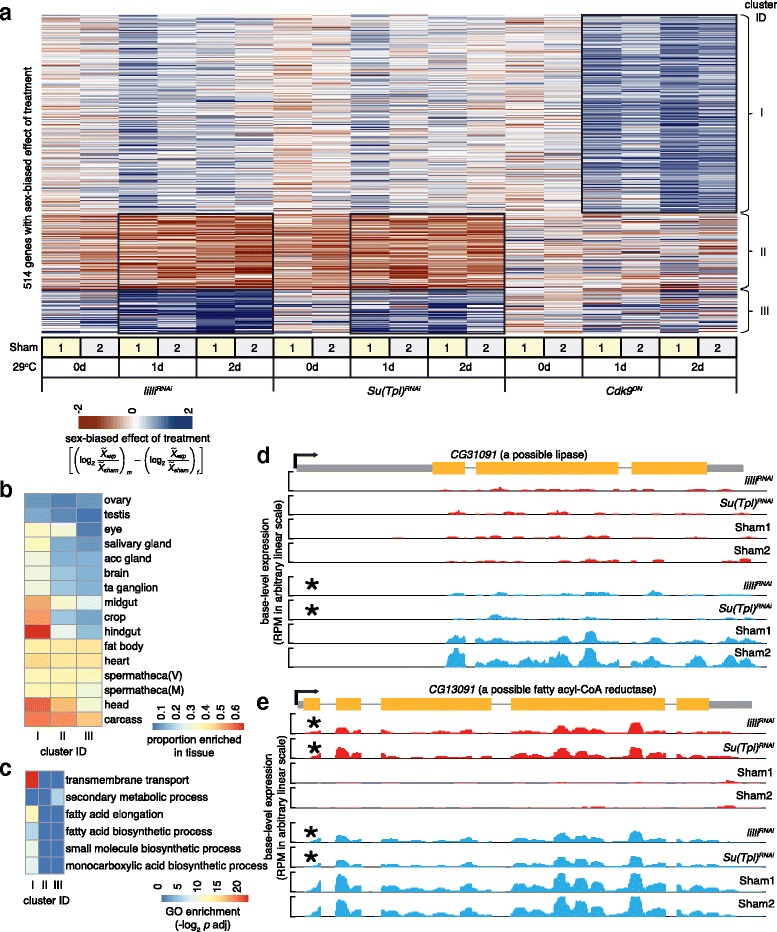
Sex-biased regulation after knockdown of elongation. **a** Heatmap of induction-dependent sex-biased effects. Clusters are labeled (*Roman numerals*). A cluster with eight genes is included in cluster II due to its small size and similar pattern as cluster II. See Fig. 2 for labeling. **b** Heatmap of enrichment for tissue specific expression in the clusters. See Fig. 3 for labeling, except m = male and f = female in the key. **c** Heatmap of GO biological process terms enriched in any of the clusters. See Fig. 3 for labeling. **d**-**e** Browser views of two examples of sex-biased gene regulation (one day after induction) are shown - *CG31091* (a possible lipase encoding gene) and *CG13091* (a possible fatty acyl-CoA reductase encoding gene). Note that these two CG names differ by placement of a single digit. See Fig. 3 for labeling, except that we distinguish female (*red*) and male (*blue*) expression

We were intrigued by the role for positive elongation factors in the expression of a large number of metabolic enzyme encoding genes expressed in the midgut, as the Drosophila midgut shows sex-biased physiology and morphology, due to factors such as the immense metabolic requirements to support egg development and the requirements for specific saturation states in lipids used for pheromone production [67–69]. Additionally, *lilli* and *Su(Tpl)* are required for external sexual morphology [51]. This raised the possibility that there could be different roles for positive elongation factors in the sexes. To explore this hypothesis, we measured sex-biased responses to gene expression treatments relative to shams for all 4792 differentially expressed genes. We then followed a k-means clustering procedure to identify genes with sex-biased patterns (Fig. 2c). We found three clusters (514 genes in total) showing a coherent sex difference in expression (Fig. 4a). Genes in cluster I showed higher expression in males relative to females following *Cdk9* ^*DN*^ treatment, suggesting a role for P-TEFb. Genes in cluster II showed reduced expression in males relative to females, while those in cluster III showed higher expression in males relative to females following *lilli* ^*RNAi*^ and *Su(Tpl)* ^*RNAi*^ treatments, but not *Cdk9* ^*DN*^. These results suggest a role for MLL in regulating sex-biased expression.

To determine where these genes with sex-biased responses might be expressed, we again compared to the FlyAtlas dataset (Fig. 4b). In addition to the digestive system, genes expressed in carcass were enriched in all three clusters. The FlyAtlas definition of carcass is the thorax and abdomen after removal of the other tissues and organs, so this category includes epidermis, musculature, and oenocytes, which perform liver-like functions in Drosophila [70]. We also found modest enrichment of genes expressed in a variety of non-gonadal tissues in the other clusters. To help determine the function of the genes showing sex-biased differential expression, we performed GO term enrichment analysis and observed that the most significant enrichment was trans-membrane transport and fatty acid elongation in genes regulated by *Cdk9* (cluster I, Fig. 4c). Genes showing sex-biased expression due to *lilli* ^*RNAi*^ and *Su(Tpl)* ^*RNAi*^ treatment were enriched in secondary metabolic processes (cluster III, Fig. 4c). Despite the lack of significant enrichment for GO terms in cluster II, we did observe sex-biased differential expression for at least a few genes encoding metabolic functions. For example, *CG31091* encodes a protein with similarity to lipases and requires *lilli* and *Su(Tpl)* activity for its male-biased expression (cluster II, Fig. 4d). This possible lipase shows highly midgut-biased expression in wild-type flies [64]. As another example, *CG13091* encodes a protein with similarity to fatty acyl-CoA reductases and low level of expression in females requires *lilli* and *Su(Tpl)* (cluster II, Fig. 4e). The putative reductase is highly expressed in wild-type fat body, heart, and carcass but not in other tissues [64]. Thus, like the overall pattern of differential expression, the sex-biased response to elongation factors was enriched in genes encoding metabolic functions.

## Discussion

### Complex components

Various positive elongation factor complexes have been described. Many of those complexes have overlapping components and complex composition is variable in the literature (Table 1) [25, 27, 31]. Therefore, genetic data can be useful for helping to determine the constituent components in biologically active complexes, or in epistatic relationships. For example, SEC contains both Lilli and Su(Tpl), while LEC and AEP contain either Su(Tpl) or Lilli (Table 1). In our expression profiles, *lilli* and *Su(Tpl)* positively regulate a nearly identical set of genes, which is consistent with them being obligatory co-factors in MLL, SEC, and EAP complexes. Our data does not support a role for LEC or AEP, although we cannot rule out the existence of these complexes. We also found that about 20% of the genes regulated by *lilli* and *Su(Tpl)* were also regulated by *Cdk9.* This is consistent with the presence of *lilli*, *Su(Tpl)*, and *Cdk9* in SEC and EAP [71]. However, we also found hundreds of genes positively regulated by *Cdk9* only. The genes specifically regulated by *Cdk9* might be dependent on P-TEFb independently of the larger order complexes.

### Metabolic responses

Promoter-proximal pausing of RNAPII is an interesting regulatory mechanism because it allows for rapid responses. RNAPII binding to the TSS, unwinding the DNA duplex and initiating transcription requires much more time in vivo (10 min [72, 73]) than elongation (about 1.5 k bases (kb) per minute [74, 75]). Such rapid responses are especially important for heat shock, pathogen defense, and proper expression of some developmentally regulated genes [36, 39, 44, 45]. We found that positive elongation factors regulate many genes encoding metabolic enzymes, especially those expressed in the midgut. Release from pausing is an attractive mechanism for rapidly producing enzymes in response to feeding. Indeed, the *Jonah* genes encode chymotrypsins in the midgut and are expressed in feeding, but not in non-feeding stages of development, such as the first 12 h of adult life when flies are maturing and using residual fat body stores [76]. In mosquitos, the orthologous genes are rapidly induced in the gut by a blood meal [77, 78]. We suggest that transcriptional elongation is an important regulator of rapid metabolic gene expression responses in adult Drosophila.

Rapid transcription also depends on gene length. Interestingly, genes positively regulated by the *lilli* and *Su(Tpl)* genes in our studies tend to have short transcripts, especially the ones with the highest level of differential expression after perturbation. For instance, most of the transcripts of chemotrypsin genes, such as *Jonah* genes and *lambdaTry*, are shorter than 1 kb, which enables transcription in less than 40 s. In general, the genes positively regulated by the *lilli* and *Su(Tpl)* genes produce even shorter transcripts than the heat shock genes (1751 versus 2231 bp). Although it may not be a prerequisite, short transcript length could be a feature evolutionarily favored by genes with rapid responses. We did not observe significantly shorter transcripts among genes regulated by *Cdk9*, suggesting that regulation of short transcripts is MLL-dependent.

### Sex-biased expression

The *lilli* and *Su(Tpl)* loci contain many conserved and highly occupied Dsx binding sites, and driving *lilli* ^*RNAi*^ or *Su(Tpl)* ^*RNAi*^ in *dsx* expressing cells results in specific switches in sexual dimorphism in the male first leg and the female genitalia, suggesting that these genes are required in both females and males, but in different locations [51]. A sex-biased role for *Cdk9* has not been described, but the locus does have a conserved consensus Dsx binding site [51]. However, we do not know if this sex- and tissue-specific role is confined to morphology, or is more general. We found that sex-biased expression changed for about 200 genes due to *lilli* ^*RNAi*^ or *Su(Tpl)* ^*RNAi*^, and for about 300 genes due to *Cdk9* ^*DN*^ *.* As was the case for morphology, the *lilli* and *Su(Tpl)* genes are required for both genes with female- and male-biased expression. This is consistent with the idea that sex-biased expression is highly context dependent and argues against strictly female- and male-biased batteries of genes that shows the same bias in all tissues. The *Cdk9* regulated genes showed a more directional effect of reduced expression in females relative to males.

Many of the genes regulated by elongation factors encode metabolic functions. There is a great deal of precedent for sex-biased metabolism. For example, egg production is energy intensive and tightly linked to nutrient availability via insulin signaling [79]. At the morphological level, the *Drosophila* midgut is nearly twice as long in females as males and is regulated by the sexual identity pathway [67]. Finally, lipids and other metabolites also show dramatic sexual dimorphism [80]. Our work suggests that some of the sex-biased gene expression in metabolic pathways is due to indirect activity of Dsx target genes such as *lilli* and *Su(Tpl)*.

## Conclusions

Turning down positive elongation factors has a global transcriptional effect. Our data suggest that Lilli and Su(Tpl) are likely to be obligatory cofactors for the expression of many genes in soma, and that Cdk9 has both shared and Lilli/Su(Tpl)-independent functions. Our data also suggest that transcriptional elongation regulation by these factors is essential for rapid enzymatic and metabolic activities during digestion, a novel category of function regulated by rapid transcriptional elongation. In addition, the differential expression in females versus males suggests that positive elongation factors play roles in sex-biased gene regulation.

## Methods

Summarized data is found in Additional file 1.  A table of key resources is provided in Additional file 2, which describes the reagent or resources used; including chemical and biological reagents, critical commercial kits, genotypes of the flies, deposited data, and software. We also provided the sources and identifiers for these resources.

### Molecular genetics

*Cdk9* ^*DN*^ transgenic lines were generated by PCR-amplifying the *Cdk9* open reading frame from *w* ^*1118*^ genomic DNA and cloned into pBluescript KS. Mutant *Cdk9* ^*DN*^, a substitution of amino acid 199 of the active site (aspartic acid to asparagine) was obtained by the megaprimer method [81] using a mutagenic oligonucleotide. The mutant DNA was cloned in the *UASp* vector [82] and injected in *w* ^*1118*^ embryos. A transgenic line with an unmapped insertion on the 3^rd^ chromosome was used in this study, but other lines show the same effect on development in the presence of various *Gal4* drivers.

### Flies

All flies were reared on the following media (per liter: 6.2 g agar, 59 g cornmeal, 59 g yeast, 5 ml propionic acid and 26 ml 99% methyl 4-hydroxybenzoate). Briefly, homozygous w*; +; P{tubP-GAL4} ^*LL7*^, *P{tubP-GAL80* ^*ts*^}^*7*^ virgin females were crossed to homozygous males with a UAS responsive transgene or homozygous males of control lines for shams. The paternal lines were *lilli* ^*RNAi*^, *Su(Tpl)* ^*RNAi*^, *Cdk9* ^*DN*^, *w* ^*1118*^, and *lacZ*. There are additional experiments in the GEO [63] series accession GSE77492 not presented here. First, we performed profiling following *gpp* ^*RNAi*^ but did not shown them in the results due to poor knockdown. Second, we used flies with an additional copy of *Cdk9* ^*DN*^, but observed a non-additive effect of two copies of *Cdk9* ^*DN*^. Third, we ectopically expressed *stand still*, but these samples were for other purposes. All the parental lines were reared and crossed at 20 °C, which also applied to their progeny. We collected adult progeny at 0 to 4 h post-eclosion, aged them for 5 to 7 days in fresh vials, and shifted to the restrictive temperature (29 °C) for 0, 1 or 2 day(s). All the flies were 7 days old at the time of dissection.

### RNA-seq

For each sample, three gonadectomized adults were dissected and crushed in 100 ul RNAlater solution (Life Technologies, Carlsbad CA) before sealing in deep 96-well plates at room temperature. Samples were homogenized with a mini-beadbeater (Biospec Products, Bartlesville OK) three times for 60 s each in the presence of 20 to 30 1.0 mm glass beads per sample (BioSpec Products, Bartlesville OK). We extracted RNA using RNeasy according to the manufacturer (Qiagen, Valencia CA), except that we added 600 ul RLT buffer and 700 ul 70% ethanol in each well. We used 400 ng total RNA in 50 ul nuclease-free water (Quality Biological, Gaithersburg MD) for RNA-seq library preparation. We strictly followed a previously reported polyA+ strand-specific RNA-seq library preparation protocol [83]. We conducted single-end 50 bp RNA-seq on the HiSeq2000 Sequencing System (Illumina, San Diego CA). Reads were mapped to FlyBase [84] release 6.04 using STAR (v2.4.2a) with default parameters [85]. The overall agreement between biological replicates was ≥ 0.88 (Pearson’s r). We also mapped the reads of all samples to the intergenic regions, which we used as a measure of background expression noise for calculating the number of expressed genes. Genes expressed at < 95% percentile of intergenic expression were counted as unexpressed [86]. All samples with 29 °C treatments were triplicated, and all samples without 29 °C treatments were quadruplicated except for female Sham2 samples (triplicated). To confirm that our results were robust to mismatched sample sizes, we used bootstrapping (100x) to randomly select three replicates among each sample set. The median number of re-identified differentially expressed genes was 96%, suggesting that the variable samples sizes have little impact on our results. Therefore, we used all replicates in this work.

### Expression analysis

For each sample, uniquely mapped read counts from HTSeq (v0.6.1p1) (--stranded = reverse) [87] were used as inputs in DESeq2 (v1.10.1). We used Normalized Read Counts as the unit of expression except for genome browser coverage plots, where we used base-level coverage of reads (generated by bedtools v2.25.0 [88]) normalized by RNA-seq library size in millions (RPM). For Normalized Read Counts, DESeq2 calculates the geometric mean (GM) of each sample, and divides the raw read counts by the GM to obtain ratios of relative expression. For each sample, we used the median ratio as a sample-specific size factor (~1 in our experiments), and normalized read counts were calculated as raw read counts divided by the size factor. Previous studies based 726 Drosophila individuals [86] demonstrated that the DESeq2 method was a high performing normalization approach relative to total counts, upper quartile, median, trimmed mean of M-values, quantile, reads per kilobase per million mapped reads, and un-normalized count data. We also used DESeq2 to calculate differential expression. For each experimental sample within the same sex and treatment, we compared experimental relative to each of the two sham genotypes. We retained genes with significantly differential expression (Wald test *p* adj < 0.05) in any of the comparisons. We further categorized these genes by their induction-dependent differential expression (log_2_ ratio of the experiment expression relative to sham) across samples. We converted “N/A” values (divide by 0) to “0” (indicating no bias) for k-means clustering. We used the parameter *k* = 18 (Fig. 2c) or 20 (Fig. 4a) based on evaluation of within-cluster sum of squares [89], and also confirmed that the major clusters were robust from *k* = 15 to 25. We used visual examination to remove clusters driven by differences between the two sham genotypes and clusters that showed weak differential expression, or responded even without induction.

We used FlyAtlas [64] microarray expression data to infer where differentially expressed genes were expressed in our experiments. We converted microarray Oligo IDs to FlyBase [84] gene IDs using DAVID (v6.7) [90]. Some genes have multiple microarray probes, and we calculated the mean signal using 1 = “enriched”, 0 = “not different”, and -1 = “depleted” as compared to the signal in the whole body. We defined enriched expression as mean > 0. Gene Ontology (GO) [91] tests were conducted in FlyMine [92]. We used a Holm-Bonferroni cutoff of *p* adj < 0.05 for GO analysis and all tests were based on the background of expressed genes in any of our samples rather than all genes. We used the pheatmap package (v1.0.8) in R (v3.2.2) to generate all heat maps of tissue specificity and GO results.

## Additional files

Additional file 1: Summary of gene expression and k-means clustering. (XLS 10240 kb)

Additional file 2: Key resources table. (XLS 55 kb)

## Abbreviations

AEP: AF4 family/ENL family/P-TEFb complex
AF4: ALL1-Fused Gene From Chromosome 4
AFF4: AF4/FMR2 family member 4
*Cdk9*: *Cyclin-dependent kinase 9*
CTD: Carboxy-terminal domain of RNAPII
DN: Dominant negative
DotCom: Dot1 complex
DRB: 5,6-dichloro-1-β-D-ribofuranosylbenzimidazole
DSIF: DRB sensitivity inducing factor
EAP: Elongation assisting proteins
FMR2: Fragile mental retardation 2
GM: Geometric mean
GO: Gene ontology
*Gpp*: *grappa*
H3K79: Histone H3 Lys 79
LEC: Little Elongation Complex
*Lilli*: *lilliputian*
*LMan*: *Lysosomal alpha-mannosidase*
MLL: Mixed-lineage leukemia 1 proteins
NELF: Negative elongation factor
P-TEFb: Positive transcription elongation factor b complex
RNAPII: RNA polymerase II
SEC: Super Elongation Complex
*Su(Tpl)*: *Suppressor of Triplolethal*
TSS: Transcription start site
